# Why human olfaction should not be modeled on theories and tasks of vision

**DOI:** 10.3389/fpsyg.2023.1244480

**Published:** 2023-09-27

**Authors:** Per Møller, Egon P. Köster

**Affiliations:** ^1^Per Møller Consulting, Bagsværd, Denmark; ^2^Helmholtz Institute, University of Utrecht, Utrecht, Netherlands

**Keywords:** olfaction, vision, explicit, implicit, ecological validity, identification, cue-target paradigm

## Abstract

In this paper we analyze some key concepts and problems in olfaction and argue that many concepts borrowed from vision are not helpful in elucidating the functions of human olfaction. This is illustrated with several examples. Olfaction is rarely in the focus of human attention. Olfaction is, compared to vision, a ‘hidden sense’, but still guides many important behaviors by way of unattended unconscious olfactory perception and implicit memory. Not all olfactory processing, however, is of an unconscious nature. Flavors, and the pleasures gained from them, are most often consciously perceived. These are experiences mostly determined by olfaction, taste, touch and chemesthesis. Our analyses lead us to conclude that olfaction should not be modeled on vision, neither conceptually nor with respect to the problems solved by the two senses. A critical examination of the ecological and physical constraints of olfaction and the other senses should be given priority. Such analyses will further our understanding of which problems are solved by the different senses and how they collaborate to guide us through the world.

## Introduction

Humans are endowed with several senses which Gibson (1966, 1979) suggested have developed to allow us to interact appropriately with the world we live in. According to Gibson, the different challenges we encounter and the different needs we have require *different* sensory systems.

Unfortunately, most human olfactory studies have not considered ecological or physical constraints of the environment. Nor is it commonplace to analyze the tasks and problems olfaction solve. Why a particular task should be solved by olfaction is rarely discussed in human olfactory studies. Rather, much work in olfaction seems to rely on a more or less explicitly stated assumption that olfaction solves many of the same problems as vision does, and that it works in roughly the same way. Of course, on fundamental levels, studies of olfaction and vision (and other senses) have in common that they should solve perceptual and cognitive problems in a dynamic environment, calling for ecologically realistic and valid experimental paradigms. From this, however, does not follow that the problems solved by the two senses are the same, nor that concepts which are useful in one sense are necessarily relevant for studies of the other sense.

Studies of all types of sensory processing would benefit from analyses akin to the approach that Marr and collaborators introduced to the study of vision (Marr, 1982; Richards, 1988). In this approach, on the so-called computational level, ecological and physical analyses of the environment and of the types of information available are undertaken. These analyses allow a determination of which tasks can be meaningfully accomplished. They also reveal when there is not sufficient information available from a particular ‘cue’ and when, therefore, information from other cues are needed to solve the problem at hand (Aloimonos and Shulman, 1989; Clark and Yuille, 1990).

The second level in Marr’s analysis of vision is a so-called ‘algorithmic level’. Which are the representations needed and which transformations of information between representations can solve the problem, i.e., make explicit the solution to the problem. Finally, in Marr’s approach, is an implementational level. How can the necessary transformations of information be implemented in neural hardware.

Marr’s approach to vision has been immensely successful in human visual studies as well as in machine vision, even though the original framework has been amended and modified in various ways.

Stevenson (2010), in a highly recommendable paper, made what he referred to as ‘an initial evaluation of the functions of human olfaction’. Stevenson identified three major classes of function: (1), a class of functions relating to *ingestion*, such as appetite regulation, breast orientation and feeding. (2), a class of problems relating to *avoiding environmental hazards* (fear or disgust related) and finally (3), problems related to *social communication*, such as reproductive issues and emotional contagion. Stevenson examined the functions he suggested with respect to ecological validity in human olfactory research. The present authors later suggested that another class of function should be added to the three classes suggested by Stevenson, namely a class of problems relating to the *‘feeling of safety’ or the feeling of being ‘at home’* (Köster et al., 2014). We will return to this problem later in the paper.

We would like to point out here that most of the problems within the four classes require no identification or naming of odors to be accomplished. Also, the importance of non-intentional (incidental) learning and implicit memory in these functions should be noted.

We will argue that much work in olfaction is misguided by an almost total neglect of the ecological conditions of the perceiving subject and that a better understanding of olfaction will follow from analyses of the functions of, and problems solved by human olfaction. Answers to questions of *which, why* and *how* certain functions are implemented by olfaction should receive more attention.

In the following we discuss some key concept of vision and examine whether they are also useful for the study of olfaction. We discuss if vision and olfaction provide different types of information about the environment and whether the two senses differ in their dependency on explicit and implicit perception and memory.

### Vision and olfaction

As already alluded to above, and expanded upon in the rest of the paper, vision and olfaction serve different roles in human perception and cognition. Anatomically, the two senses are organized in completely different ways. Visual information is transmitted from the eyes to the brain in a contra-lateral fashion, whereas (most) olfactory information reaches the brain ipsilaterally (Paxinos and Mai, 2004). Vision is based on three types of receptors in the eye, whereas it has been estimated that human olfaction relies on hundreds of different receptors in the nose (Paxinos and Mai, 2004). Wiring in the brain of the two systems is also very different, with systems in limbic structures having earlier access to olfactory information (Van Essen et al., 1992; Paxinos and Mai, 2004; Rolls, 2021, 2023). These different architectures of the human visual and olfactory systems suggest that the two systems solve different perceptual and cognitive problems.

Köster made a comparison between vision and olfaction for a number of important perceptual and cognitive problems (Köster, 2000). We refer the reader to this paper rather than discussing it here, except for the conclusion of the comparison that the properties of the two systems are very different with respect to: strict inter-subjectivity, inborn properties, directional perception, relative perception, intensity perception, adaptation, and being in focus of attention.

Vision provides information that allow us to solve spatial tasks. The layout of the environment and the distances and sizes of objects can be inferred from visual information, as can the directions and movements of objects, as well as our own movements in the environment. We move safely around in our environment without bumping into things or being hit by a bus when crossing the street. The locomotion problems faced by blind people clearly demonstrate the importance of vision for *spatial tasks.*

Vision also provides information about the shape of *objects.* The physical objects in the world reflect light into our eyes that allow us to infer their distances and properties. The concept of a ‘visual object’ is meaningful and it is a direct representation of the physical objects in the world with shapes and material properties.

Location, movement, and identity of objects are obviously important properties of the environment and information about these properties can be obtained by visual processing. At the core of the argument, vision provides information about ‘what is where’, as succinctly expressed by Marr (1982). This dictum has later been amended in various ways, but that is not important for the arguments we want to make in this paper.

Vision *can* provide information about ‘what is where’. But what about the other senses? Olfaction, e.g.? Is it possible to obtain reliable information about locations and identities from olfactory information?

The enormous progress in our understanding of human (and machine) vision since the late 1970’s is based on ecological and computational analyses. Detailed analyses of image formation and reflectance functions (Gibson, 1966, 1979; Marr, 1982; Horn, 1987; Richards, 1988; Koenderink, 1990) have been instrumental for this progress. A description of image formation is crucial for understanding the information available at the eyes, as are computational theories to demonstrate which transformations and representations are necessary to accomplish a given task.

Olfactory information is provided from substances from objects which can activate olfactory receptors in the nose. Ecological and physical analyses of diffusion and convection would seem to be crucial in understanding the information available at the olfactory receptor level. The fact that many objects do not smell should also be included in reflections about the functions of human olfaction. Unfortunately, such considerations are not ‘top of mind’ in much olfactory research. In a ‘normal’ environment the olfactory stimuli humans receive is a very complex mixture from many physical objects. Olfaction is a synthetic sense, i.e., individual ‘molecular smelling features’ of the hundreds of smelling molecules are not consciously available. Segmentation into separate objects and ‘reconstruction’, i.e., determining *which* objects are present, are therefore next to impossible tasks for olfaction. In real life.

Of-course, olfactory notes of well-known objects such as bananas or coffee can be easily detected, but based on olfactory information alone, all we can know is that bananas or coffee is present somewhere in the immediate environment.

Despite the absence of any serious considerations of ecological importance, there are recent examples of olfactory work ‘inspired’ by visual problems and concepts. We will briefly discuss two papers describing such work and why we find them misplaced. We will also comment on work on olfactory identification, which is probably the most studied topic in olfaction.

## Examples of important perceptual problems solved by vision, which have also been investigated in olfaction

### Way finding

Humans can *effortlessly* find their way around in the environment by means of visual information. Can olfaction also solve way-finding problems for humans? After all, dogs are very good at following a trace by means of olfactory cues. This question was investigated by Porter et al. (2007). These researchers laid down a 10-meter-long trail of chocolate essential oil in a grass field and had 32 blindfolded subjects track the scent. The 10-meter-long trail should be tracked in 10 min (*sic*!) and each subject got 3 chances to track the scent. Two-thirds of the subjects finished the task. Thrilled by these results the researchers concluded that “These findings reveal fundamental mechanisms of scent-tracking and suggest that the poor reputation of human olfaction may reflect, in part, behavioral demands rather than ultimate abilities” (Porter et al., 2007).

Can we conclude from these results that olfaction aids humans in wayfinding? A 10- meter-long trail could by some subjects be tracked in 10 min. Using visual cues, the task could be accomplished in about 5 s. According to the authors the problem of olfaction’s ‘poor reputation’ reflects behavioral demands rather than ultimate abilities. In other words, they argue that olfaction *has* the ability to help humans find their way around in the environment. Unfortunately, humans are extremely bad and slow at it.

Few people would disagree that humans move around on two feet with the nose 1,5–2 meters above the ground. As opposed to what is the case for dogs, who have their nose very close to the ground, this means that scents on the ground are not as effective at stimulating human noses as they are for dogs. Also, in outdoor environments the wind blows and scatters scents to an extent that over only short periods of time, all that can be extracted by the human olfactory system is that some source producing a certain smell is present somewhere in the immediate environment. These simple ecological and physical considerations would seem to rule out olfaction as a contributor to wayfinding. It is re-assuring that these theoretical considerations are confirmed by the experimental results, even though the authors seem to conclude the opposite.

This analysis should not be understood as a claim that smells cannot be used as rough indicators of locations in environments with aerial stability. Some examples of how smells can be used to help people find around in homes for the elderly have been described (e.g., Köster et al., 2014; Cameron et al., 2021). But outside, where the wind blows and odorous materials are constantly mixed, animals with their noses 1.8 meters above the ground would be in dire trouble by relying on olfactory signals to guide wayfinding.

### White odors

Weiss et al. (2012) claimed to have created “olfactory white” or laurax as they called it. They showed that by selecting odorants that were well spread over both perceptual and physicochemical spaces and reducing them to about equal intensities, different concoctions of groups of 30 odorants could be made that were more difficult to discriminate from each other than when such mixtures contained fewer components. They claimed that such mixtures, composed of a huge number of components formed the olfactory equivalent of white light and white noise. This search for an “olfactory white” was inspired by the existence of a “visual white” in vision. White light (a broadband light) is a mixture of narrowband lights and can be created by infinitely many combinations of “chromatic lights,” i.e., lights with color appearance (blueish, reddish, greenish, etc.).

Unfortunately, discriminability of their different mixtures of thirty components remained still well above chance. Other attempts in the paper by Weiss et al. (2012) were equally unsuccessful at supporting the idea of the existence of an olfactory white.

The idea behind “olfactory white” seems to be that what we have in vision, we also have in olfaction. Unfortunately, ecological considerations about why it would be advantageous to have an “olfactory white” are not discussed in the paper. In vision, ecological and computational analyses, in the sense of Gibson (1979), Marr (1982), and Richards (1988), demonstrate why white light is important. Broad-band lighting is essential for color constancy, the phenomenon that an object has roughly the same color under different lights. When light is reflected from the surface of an object, a certain amount of the different wavelengths hitting the object are reflected. How much of a certain wavelength is reflected is determined by the reflectance function, which is a property of the object. If an object is viewed under a single wavelength (or a very narrow band lighting) the reflected light will consist of this single wavelength and the object will appear to have the color of the light and not represent the reflectance function of the object. Color constancy has broken down in this case. Under different broadband lightings, an object has the same color even though there are large variations in the light reflected from the object. Your car has the same color in bright sunshine as it has under cloudy skies or street lightning in the night. Color constancy is important for visual object recognition and for visual searching for objects.

White light and differences in brightness are important for other accomplishments of the visual system than color constancy. Most of visual motion perception is driven by luminance contrast, and stereoscopic extraction of shape is also dependent on luminance contrast. The same goes for inferring ‘shape from shaping’ (e.g., Marr, 1982; Horn, 1987; Richards, 1988).

“White light” plays a key role in spatial perception and visual motion and shape perception. These are all properties of our world of which olfaction carries very little, if any, information. What could be the ecological role of “olfactory white”? Unfortunately, the authors leave this challenge as an exercise for the reader.

One might perhaps also argue that if cases of olfactory white existed, they might not have been noticed for their lack of specificity and would certainly not be cultivated like wine, roses, and coffee. Furthermore, it is perhaps good to remember that if olfactory white existed permanently, people would not perceive it, because they would probably be completely adapted to it.

Weiss et al. (2012) end with the hope that olfactory white, notwithstanding its absence or extreme rarity in the real world, can serve a similar function as white light and white noise have served in the neurobiological study of vision and audition. The present authors share this hope in as far as it might clarify the essential differences between the mechanisms involved in senses as different as vision or audition on the one hand and olfaction on the other, instead of just pre-supposing that all senses are essentially similar and that olfaction must, therefore, function in the same way as vision.

### Odor identification

Odor identification is probably the topic that has been most scrutinized by olfactory scientists. Very many papers have been published on this subject. As we discussed above, object identification is clearly an important problem in vision. As can already be suspected from the term ‘odor identification’, there might be an assumption that the odor that arrives at the nose derives from a well-defined *physical object*, like is the case in visual identification. In olfaction this is only the case in the laboratory when subjects are presented with well-defined object-odors from bottles or olfactometers. In real life, the odors that hit the nose are complex mixtures of odorous materials emanating from *many* objects, which in most cases cannot be separated since olfaction is a synthetic sense.

Imagine a normal day where you wake up in the morning in your bedroom. From the bedroom you move to the kitchen and after coffee you make it into the bathroom. All these different rooms/environments smell differently, but you normally do not notice the different smells. You can, of-course, direct *attention* to the smells in the different rooms by sniffing around and thereby realize that they do indeed smell differently, but without this attentional effort you will not normally notice the different smells of the rooms. A smell is normally not consciously noticed, unless it is unexpected in a certain environment.

After breakfast you decide to bike to the local food market. This is a nice tour that takes you through a small forest before you have to cross a major road with heavy traffic. Unless something is out of order in the forest, you will not notice the particular smell of trees and earth etc. A smell of diesel, however, will immediately attract your attention since this smell does not fit with the forest environment.

To cross the road safely, you use visual information to judge the distance to, and speed of, the cars you have visually identified. The path you follow is guided by visual information. Olfaction plays no role in solving these problems.

Only when shopping for food in the market you consciously smell the particular food products you are interested in, by picking them up and smelling them to evaluate their quality. You also use the sense of touch to evaluate textural properties. Flavor and texture quality cannot be inferred from visual information alone. Color and shininess etc. can be important quality markers of foods, but most people will smell the foods they buy, if it is possible. Also, it is commonplace to be offered taste samples when shopping for cheeses and wines and many other food products. Vision cannot provide the necessary information needed to make an informed decision in these cases.

In the very many studies of olfactory identification (e.g., Cain, 1979; Cain, 1982; De Wijk et al., 1995; Cain et al., 1998; Cleary et al., 2010; Croijmans and Majid, 2015) workers often remark on the difficulty people have in identifying even well-known common odors. However, it is important to notice that olfactory research has been run with restricted, highly selected samples. So far, our psychological and psychobiological knowledge about olfaction has been generalized from research prevalently run on participants belonging to WEIRD (western, educated, industrialized, rich and democratic) societies and, even within these populations, convenience samples of young adults (undergraduates in psychology) have been typically investigated (Henrich et al., 2010). Thus, our understanding of human perception in general and olfactory perception, in particular, is extremely biased and probably unsound in terms of cultural and ecological generalizability. There is a huge paradox here that the (Western) populations in which olfaction has been most studied are the ones in which elites appear most depreciative and even repressive toward the sense of smell. Thus, research in cultural groups (or less studied subgroups in WEIRD societies) in which odors are part of the common experience in adaptive life-sustaining or expert activities may change our view of olfactory functioning and functions in humans at large. For example, Majid and coworkers have demonstrated that hunter-gatherers’ olfactory cognition is different from that in WEIRD people (e.g., Wnuk and Majid, 2012; Majid and Kruspe, 2018). Thus, with the proviso that our discussion here of odor identification is based on data from WEIRD people, it has been found that of the many thousands of odorants and odorant mixtures that we meet, most WEIRD people can identify and name perhaps only about 50% of the most common odors presented to them, unless higher level and explicitly functional categories (e.g., edibility) are involved (e.g., Engen and Pfaffmann, 1960; Engen, 1991; De Wijk and Cain, 1994; Cain et al., 1998; Zarzo, 2008).

Although people, especially those in urban western and westernized societies, are notoriously bad at identifying odors by name (Richardson and Zucco, 1989; Schab, 1991; Herz and Engen, 1996; Cain et al., 1998), they often easily remember where they smelled an odor encountered before and use this information to deduce what might have been its source in that situation.

Not only is odor identification very bad, it is also very slow and not consistent. In an experiment by Cameron et al. (2016) the same odor was presented twice and only about 40% of the odors named by subjects were given the same name on both trials.

So, why is there so much (scientific) attention to human odor identification, when everybody seems to agree that humans are very bad at it? In discussions with colleagues one of the authors have been confronted with the argument that it is exactly because humans are so bad at it, that we should disentangle the ins and outs of it. This is an argument that seems to rest on an *assumption* that we *should* be good at odor identification. This kind of reasoning is not very fruitful, we think. Rather, if we are not good at a specific task using olfactory input, this task is probably not very important for understanding olfaction.

Standing on toes like a ballet dancer is indeed a difficult task, but understanding how and why this is possible will not bring us very much closer to understanding why we have feet. Some ecological analyses of what having feet allows us to do seems to us to be a more fruitful starting point for investigations of the functions of feet.

Then, why has so many resources been invested in studies of human odor identification? This is more a sociology of science question than a proper olfactory science question. We can only speculate since we have not studied this historically and sociologically.

Odor identification is an integral part of a number of commercially available odor test kits^1^ and people who have bought these kits might have thought that the presence of an odor identification task in their kit is therefore an important task to execute and publish about. Another reason might be that it is easy to administer odor identification tasks. Or it might be that many olfactory scientists model their thinking on vision. Or it might be that humans are not at all bad at odor identification as we have argued above. Maybe the seemingly weak human ability to identify odors is a result of weak experimental paradigms that have been used to investigate it? This has recently been argued by Pierzchajlo and Olofsson (2023) who have introduced a so-called cue-target paradigm for the investigation of odor identification.

### Cue-target paradigms in olfaction

Pierzchajlo and Olofsson (2023) suggest that human olfaction should be understood in terms of its reliance on top-down processes from visual or verbal information. They suggest that visual or verbal contexts *generate predictions of odor qualities*.

Furthermore, they claim that a fundamental role of olfaction is to evaluate these predictions. In their view, thus, odor identification is a fundamental task for human olfaction. Olofsson and coworkers have pursued these ideas in a range of papers (Olofsson et al., 2012, 2013, 2014; Olofsson, 2014; Olofsson and Gottfried, 2015).

They make a big deal out of the distinction between *un-cued* and *cued* odor naming, which they refer to as naming and identification, respectively. Cued odor naming means that a word/name or a picture of an object is shown when smelling an odor and the task is to choose the correct name or picture of the object giving off the smell.

In the cue-target paradigm used by Olofsson and coworkers, a visual or verbal cue (priming stimulus) is presented briefly before the odor (target) and the reaction time task is to respond ‘yes’ or ‘no’ to whether the cue and target represent the same ‘object’. The results of such experiments on cue-target matching are that congruent trials (correct answer is ‘yes’) are always faster than non-matching trials. To explain their results, they posit that *predictions* from higher-level brain areas (verbal, e.g.) attempt to predict inputs occurring at lower-level olfactory levels. i.e., that verbal (or visual) stimuli are translated into olfactory representations and compared to the actual activation in lower sensory circuits (Why such a scheme would produce slower reaction times when cue and target are non-matching are not quite clear and would seem to require some extra specification of the actual implementation of the prediction and comparison process).

An alternative explanation of the cue-target results could be that the cue sets up a search for particular notes in the target odor. Olfaction is highly prone to verbal suggestions, as anybody who has ever participated in a wine-tasting exercise has experienced, when notes that were previously hidden are suddenly clearly present after the sommelier has described them. This is an attentive effect, based on search for memorized notes.

Nevertheless, from their results, the authors conclude that odor identification operates at a high level and is fast. And they use these results to ditch the usefulness of ‘novelty’, which is a key concept in the so-called MISFIT theory (Köster et al., 2014), for the functioning of human olfaction.

There are a few questionable aspects of the cue-target paradigm as used here.

In the cue-target paradigm, subjects *confirm* that the smell corresponds to the cued image or word. Therefore, this task is a confirmation task, not a real identification task. The problem (from an ecological or survival point of view) is why bother with confirming the identity of an object that has already been identified?

As far as the *prediction* goes it is based on explicit conscious priming by way of cues subjects are fully aware of. This is contrary to the implicit predictions posited by other recent work (Köster et al., 2014) which are generated by incidental learning of associations between situations, environments, and odors.

Furthermore, prediction without more than one possible outcome is not very meaningful. A non-trivial prediction requires that other possible outcomes than the one which *is predicted* have significant probabilities of occurring. Think of a prediction of the weather tomorrow. In non-trivial predictions we want to know the combination of temperature, sunshine, rain, wind etc. These parameters together produce a whole host of possible outcomes, most with non-zero probabilities of occurring.

In the cue-target paradigm as described above only one visual object or verbal object name is used to ‘predict’/prime the target odor. Furthermore, only well-known odors with a name or odors which can be meaningfully depicted by an image of an object are meaningful stimuli. This leaves all the possible odors humans can perceive, and which we encounter everywhere, outside the scope of this paradigm.

The functional roles of odors unknown to a particular subject, which is the majority of odors in the world, therefore, cannot be studied within this framework.

Olfaction can smell around corners as opposed to vision which relies on un-obstructed reflected light from objects into the eyes. That is, odors can be detected without the odor source being in sight, which it has to be in the cue-target task.

That only a *well-known* odor emanating from a *visible* well-defined object can be used in the cue-target paradigm does not necessarily invalidate the paradigm as a *tool* to further our understanding of the functions of human olfaction, but the processes implicated in the paradigm clearly cannot be of much importance in everyday life. Results generated from this paradigm, thus, has next to no ecological validity.

Even when confining oneself to an artificial lab environment where stimuli can be well specified and controlled, it is necessary to seriously analyze why the task/problem under investigation is important. In the present case, arguments should be put forward explaining why odor identification is an important function of human olfaction. As argued above, we are not convinced that this is the case.

Of-course the senses work together to solve real problems, but generally not by them individually solving the same task, e.g., object identification. An example of cooperation between olfaction and vision occurs when a rotten smell is detected in a cupboard or refrigerator. Once detected by the nose, we do not sniff around in the refrigerator, but use vision to search for the source of the rotten smell. Then, when vision has detected a food that might have gone off, we sometimes confirm this by picking it up for closer olfactory scrutiny.

Humans are very bad at talking about olfactory experiences and our olfactory vocabulary is very limited compared to the vocabulary we have for visual experiences. We argue that odor identification is not an important task for olfactory perception and that olfactory properties are not there to be *talked* about. What *can* be talked about are the dangers and pleasures that olfactory perception signals, not the olfactory experience itself.

Even though odor identification does not seem to be ecologically important the many studies of it have revealed several interesting and important results. It has been shown that deficits in olfactory identification or naming can be an indication of neurodegenerative diseases such as Alzheimer’s disease or Parkinson’s disease and that it might be able to dissociate between neurodegenerative disease and depressive disease (e.g., Hawkes and Doty, 2009). These are important results that are definitely worth pursuing.

The three problems discussed above exemplify why olfaction should not be ‘modeled’ on vision. Not all concepts from vision are useful for understanding olfaction (Köster et al., 2014; Barwich, 2019; Bochicchio and Winsler, 2020; Barwich and Smith, 2022).

## Examples of important olfactory problems which vision has not much to say about

As discussed above, olfaction is crucial for at least 4 different classes of function (1) a class of functions relating to *ingestion*, (2) a class of problems relating to *avoiding environmental hazards*, (3) problems related to *social communication and* (4) providing people with *a ‘feeling of safety’* or ‘*the feeling of being at home’.*

Olfaction is paramount in creating the flavor perceptions of the food and drink we consume. Mixtures of the 5 basic tastes cannot create the flavor of orange, coffee etc., and the enormous variation in possible flavor perceptions mainly results from (retronasal) olfactory perception (e.g., Stevenson, 2009; Prescott, 2012; Doty, 2015).

Other senses also influence flavor perception. Mouthfeel, perception of food texture, and chemesthetic perceptions (hot spices) obviously add dimensions to food and drink perception. Even vision and audition sometimes play a role, as has been demonstrated in recent work in the new field of Neurogastronomy (e.g., Shepherd, 2012; Spence, 2017; Spence, 2020b), but most often, effects on flavor perception from other senses than olfaction, taste and chemesthesis are ‘second order’. The shape and color of foods can only slightly change their flavor.

In most cases described in the literature, visual and auditory input does not fundamentally change the flavor as determined by olfaction and taste. A very interesting experiment on the color of wine, however, *might* suggest that this is not always the case. Morrot et al. (2001) demonstrated that the very same wine (a white wine) was perceived very differently when the experimenters had added red dye to it, having it appear as a red wine. More descriptors used for red wine characterization were used for the (white) wine when it visually appeared to be a red wine. This result might be an example of an ‘attentive top down’ effect where the color of the wine aids in search for particular olfactory notes in the wine, or it might be a fascinating example of how expectations formed by information from one sense can fundamentally change perception by another sense (Brochet and Dubourdieu, 2001; Morrot et al., 2001).

Another related problem is the so-called food pairing problem. Which combinations of foods and drinks go particularly well together and enhance the pleasure of the flavor of one or both? Vision probably does not have much to contribute to understanding this problem. Just imagine if a certain pairing of a food and a wine would be fundamentally changed by blindfolding the eater. Our conjecture is that in most cases this would be immaterial.

Cooks and sommeliers have knowledge about good pairings, but the problem is not well-understood by the cognitive sciences (Møller, 2013; Spence, 2020a; van Bergen et al., 2022; Durrieu et al., 2023).

More work on the food pairing problem might also contribute to a better understanding of ‘the nature of pleasure’ (Berlyne, 1970; Berns, 2005; Lévy et al., 2006; Kringelbach and Berridge, 2010). What does it take to produce and experience (food) pleasure? What is the role of ‘collative’ properties (e.g., novelty, perceived complexity) in pleasure, etc.?

Olfactory stimuli can be perceived in two fundamentally different ways. Orthonasally where stimuli get access to olfactory receptors via the nostrils, and retronasally where stimuli reach olfactory receptors via the nasopharynx connecting the mouth and the nose (Rozin, 1982; Small et al., 2008; Shepherd, 2012).

Anticipatory behavior is guided by orthonasal olfaction, whereas retronasal olfaction contributes to consummatory behavior via olfaction’s contribution to flavor perception. These two routes to olfactory perception do not seem to have a visual analog.

In everyday life, incidental learning is much more important than intentional learning, especially in odor and taste memory where people are never asked to learn intentionally. Incidental learning is responsible for (food) preference formation and thereby for much of eating behavior (Schaal et al., 1998; Mennella et al., 2001; Hausner et al., 2010; Remy et al., 2013; Hetherington et al., 2015; Nicklaus and Schwartz, 2019; Ustun et al., 2022).

We claim that implicit memory plays a key role in olfaction and that explicit recollection/memory of an odor does not have much ecological importance, even though, as with so many other laboratory tasks, explicit memory and identification *can* be learned up to a not very impressive level. There seems to be some agreement among olfactory scientists that smells are rarely in the center of attention in the world outside the laboratory (Sela and Sobel, 2010; Köster et al., 2014; Bochicchio and Winsler, 2020). This is unfortunately not reflected in much recent work in olfaction. Also, the concepts of bottom-up and top-down processing are sometimes treated in a very simple-minded way.

The discussion above has focused on fundamental differences between vision and olfaction especially concerning the tasks they solve and the lack of ecological and computational analyses of olfaction. We have described a number of perceptual problems solved well by vision but very badly by olfaction and vice versa.

## Olfaction: what is it good for?

As we discussed above, olfaction is crucial for at least 4 different classes of function (1) a class of functions relating to *ingestion*, (2) a class of problems relating to *avoiding environmental hazards*, (3) problems related to *social communication and* (4) providing people with *a ‘feeling of safety’* or ‘*the feeling of being at home’.*

These 4 types of function are all fully operational without any input from other senses. This is not to suggest that olfactory information is not integrated with other types of sensory and cognitive information to optimize behavior in various tasks, but rather to state the point that olfaction takes on way more important roles than the identification confirmation proposed by Pierzchajlo and Olofsson to be of utmost importance in human olfaction.

Olfaction is seldom in the focus of attention and therefore awareness of odors is rare. This means that either explicit olfactory perception and memory is not important for humans, and/or that olfaction in most cases guides human behavior by way of unattended unconscious olfactory perception and implicit memory. Some examples of the importance of unattended or unconscious olfactory perception are the following:

Body odors can modulate the evaluation and selection of mates (e.g., Schleidt et al., 1981; Schaal and Porter, 1991; Havlicek and Roberts, 2009; Wyatt, 2014; Jaworska et al., 2017). Odor experience created by the mother largely shapes infant odor preferences at the earliest stages of development (e.g., Schaal et al., 1998, 2000; Mennella et al., 2001) and directs responsiveness toward the mother’s breast (e.g., Macfarlane, 1975; Doucet et al., 2007). The formation of food preferences is initiated already in the fetal state and continues after birth by repeated exposure to foods. Olfaction plays a key role in flavor perception and is therefore important in forming the memories constituted by food preferences, which are in many aspects implicit in nature (Schaal et al., 1998, 2000; Hausner et al., 2010; Hetherington et al., 2015; Nicklaus and Schwartz, 2019; Ustun et al., 2022).

Many authors have shown that unattended and not consciously perceived odors influence *social judgments* (Cowley et al., 1977; Sabri et al., 2005; Li et al., 2007) in which it could be shown that odorants had a greater effect on perception when they were not consciously perceived, and *mood* (Van Kirk-Smith et al., 1983; Zucco et al., 2009) and *behavior*, as shown in experiments in which the odor of floor cleaner from an unnoticed bucket invoked people to clean the crumbs from their table (Holland et al., 2005).

Many other examples of effects of not consciously perceived odors have been described on memory, emotion and as a driver of consumer behavior (Chen and Havilland-Jones, 2000; Havlicek et al., 2005; Gelstein et al., 2011; Sorokowska et al., 2012; Sorokowska, 2013; De Groot et al., 2014, 2017; Iversen et al., 2015; De Luca and Botelho, 2019).

In memory studies it has been demonstrated that unattended and not consciously perceived (not subliminal) odors are very effective, whereas they may lose their effectivity by becoming consciously perceived or identified and nameable (Degel and Köster, 1999; Degel et al., 2001; Köster and Degel, 2001).

The examples given above suggest that olfaction for many important tasks acts as a hidden sense. Incidental (non-intentional) learning and implicit memory seem to be far more important than intentional learning and explicit memory and verbalized odor identification.

In our view, olfactory studies would benefit from more systematic analyses of the ecological constraints it operates under, and from a critical examination of the tasks it contributes to. It is sobering to remember that olfaction is only one of many human senses that allow us to perceive the world. The senses produce different phenomenological experiences, but they also cooperate to guide us safely through our environment.

It is a challenge to develop rigorous ecologically valid experiments. The reductionism of laboratory experiments can be useful for some problems, but it is always important to relate such work to the world outside the laboratory.

## Conclusion

We conclude that olfaction is an ‘intimate sense’ that provides much information which cannot be extracted by other senses. Vision and olfaction mostly serve different roles in guiding humans in their environment. Olfaction is seldomly in the focus of attention. Unconscious perception and implicit memory play key roles in the functions of olfaction and concepts developed in vision science are most often not helpful in understanding the role of human olfaction. A critical examination of the ecological and physical constraints of olfaction should be given priority. In the light of the analyses in this paper we conclude that olfaction should not be modeled on theories and tasks of vision.

## Author contributions

The authors contributed equally to the writing of this paper. Both authors listed have made a substantial, direct, and intellectual contribution to the work and approved it for publication.

## Footnotes

^1^Sensonics: www.sensonics.com; Burghart: https://smelltest.eu/en/burghart-sniffin-sticks-burghart-smelltests/.
